# Effects on Lower Extremity Neuromuscular Control Exercises on Knee Proprioception, Muscle Strength, and Functional Level in Patients with ACL Reconstruction

**DOI:** 10.1155/2019/1694695

**Published:** 2019-11-15

**Authors:** Defne Kaya, Hande Guney-Deniz, Cetin Sayaca, Mahmut Calik, Mahmut Nedim Doral

**Affiliations:** ^1^Department of Physical Therapy and Rehabilitation, Faculty of Health Sciences, Uskudar University, İstanbul, Turkey; ^2^Department of Physical Therapy and Rehabilitation, Faculty of Health Sciences, Istinye University, İstanbul, Turkey; ^3^Department of Physical Therapy and Rehabilitation, Faculty of Health Sciences, Istanbul Okan University, İstanbul, Turkey; ^4^Department of Physical Therapy and Rehabilitation, Faculty of Health Sciences, Hacettepe University, Ankara, Turkey; ^5^Department of Orthopaedics & Traumatology, Faculty of Medicine, Ufuk University, Ankara, Turkey

## Abstract

**Objective:**

The purpose of this study was to determine the effects of lower extremity motor control exercises on knee proprioception, muscle strength, and functional level in patients with anterior cruciate ligament reconstruction (ACL-R).

**Materials and Methods:**

Thirty-two of the 57 patients with ACL-R using tibialis anterior allografts were divided into two groups. Group I: lower extremity motor control exercises were added to the standard rehabilitation program. Group II: standard rehabilitation program was applied. Effects of lower extremity motor control exercises on quadriceps and hamstring muscles strength, knee joint position sense, and hop test were evaluated.

**Results:**

There were no differences in muscle strength and endurance of the quadriceps and hamstring between the operative and nonoperative sides in Group I (*p* > 0.05) while there were significant differences in strength of the quadriceps and hamstring between the operative and nonoperative sides in Group II (*p* < 0.05). There were significant differences in the endurance of the quadriceps and hamstring and knee joint position sense at 15°, 45°, and 75° between the operative sides of the patients in both groups (*p* < 0.05).

**Conclusions:**

The neuromuscular control exercises program was found to be more effective in reducing the difference in strength while the standard program was found to be more effective in reducing the difference in endurance between the operated knee and the other knee. This study revealed that neuromuscular control exercises should also be used to improve knee proprioception sense following ACL-R.

## 1. Introduction

A successful rehabilitation program should benefit the patients to return to an active lifestyle and preinjury levels. Outcomes of the numerous rehabilitation programs such as standard, aggressive, accelerated, home-based, supervised, and intensive programs in the literature have been discussed [1–7].

Rehabilitation programs for anterior cruciate ligament reconstruction (ACL-R) include a lot of parameters such as regaining the early full passive knee extension, immediate range of motion (ROM), proprioception, quadriceps and hamstring strengthening, and fast return to normal daily living activities and sports [1–4].

Asymmetries in physical performance such as hopping, jumping, landing, loading, and movement pattern between the reconstructed and uninjured (another side) legs were described in the patient with ACL-R [8, 9]. This asymmetry can be seen for several years after ACL-R surgery [9, 10], and it must be treated to protect the joints from abnormal loading, to prevent secondary injuries, and to develop correct movement patterns. Neuromuscular control exercises such as squat, stairs descending and ascending, and landing should be added in a rehabilitation program to enhance bilateral symmetry and motor control of the leg.

To the best of our knowledge, there have been no studies evaluating the mentioned outcomes of the neuromuscular motor control exercise programs after ACL-R with tibialis anterior tendon allograft. Therefore, the purpose of this study was to assess the effects of motor control exercises on muscle strength, proprioception, and functional level after ACL-R with tibialis anterior tendon allograft. The hypothesis of the present study was that nonathletic patients who had undergone a standard rehabilitation program with lower extremity motor control exercises had better outcomes compared to patients who had undergone a standard rehabilitation program.

## 2. Materials and Methods

### 2.1. Study Design

This randomized-controlled study with two-year follow-up was designed to evaluate the effects of the standard rehabilitation program with motor control exercises on muscle strength, proprioception, and functional level in patients with ACL-R.

### 2.2. Participants

Patients were recruited between January 2016 and December 2018. Fifty-seven patients who had undergone primary ACL-R using tibialis anterior tendon allograft at Hacettepe University were invited to participate in the present study. Patients were eligible for the study if they (a) were from 14 to 55 years of age, (b) were male sex, (c) had ACL-R surgery at least before two years, and (d) had no previous history of knee surgery. Patients who underwent meniscal repair/meniscectomy/chondral surgery were also excluded from the study. The present study protocol was reviewed and approved by the Institutional Review Board of Yedikule Chest Diseases and Thoracic Surgery Training and Research Hospital Institutional Review Board (29012015/617). The written informed consent form was signed by all patients before the study, which was conducted according to the ethics guidelines and principles of the Declaration of Helsinki.

Patients were divided into two groups using the software program (Random Allocation Software®) by the fourth author (MC) who was blinded to the assessment and group details. Group I: lower extremity neuromuscular motor control exercises were applied into the standard rehabilitation program. Group II: standard rehabilitation program was applied.

### 2.3. Blinding

The assessor of muscle strength, proprioception, hop distance, and functional scores was also blinded to the patient's identity and rehabilitation groups/protocols. The first author who designed and carried out the rehabilitation program and followed up the patients was blinded to tests. The third author who carried out the rehabilitation program and followed up the patients was also blinded to tests. All tests were performed by the second author (HGD) who was blinded to the groups.

### 2.4. Interventions

The standard rehabilitation program was applied to all patients of both groups. The standard rehabilitation protocol was performed in light of the accepted orthopedic rehabilitation textbook [11]. Neuromuscular control exercises were started at the third week after ACL-R surgery for patients in Group I [12, 13].

All patients underwent a standard rehabilitation program from the first day to the end of the second week after ACL-R surgery. All patients performed exercises in their home. Follow-up of the rehabilitation program was performed by the first author (DK) and the third author (CS) at the third week, sixth week, third month, sixth months, a year, and two years following surgery. The rehabilitation progress was determined by the functional level of patients. Follow-up checklist of the exercises was used.

On the first day after surgery, all patients underwent a standard rehabilitation program and tolerated weight-bearing mobilization with crutches. The movement of the knee was set to the extension/flexion of 0°–90° from the first days after surgery to the third week. The restrictions such as active knee terminal extension exercises were lifted after the 6^th^ week after surgery. Running was recommended at the 13^th^ week and agility and sports training at the 18^th^ week. Plyometrics and agility exercises were started from the 20^th^ week to the 24^th^ week. Return to sports was not allowed before nine months after surgery.

Neuromuscular control exercises were added to the rehabilitation program for patients in Group I (see Table 1). Single leg stance, balance reach leg and balance reach arm exercises, lunges (all directions), step-up (all directions) on the other side of the patients, and bilateral squat were started at 3 to 4 weeks after surgery. Single leg stance, balance reach leg and balance reach arm exercises, lunges (all directions), step-up (all directions), step-down (all directions), one-legged squat, box heel touches (all directions), and single leg pelvic bridge on the operated side of the patients, and ball exercises during athletic position were started at 5 to 8 weeks after surgery. Single leg straight leg dead lift, sumo squat, and weights added to all exercises were performed at 9 to 12 weeks.

### 2.5. Outcome Measures

All tests were performed at two years after surgery. Subjective tests, knee muscle strength tests, knee joint position sense tests, and one-legged hop test were done by the second author (HGD).

All patients executed 5-minute warm-up and then learned to exert peak torque in a practice session before proper data collection. The warm-up consisted of four submaximal contractions at each speed test.

#### 2.5.1. Subjective Parameters

The pivot shift, anterior drawer, and valgus stress tests were applied to control the laxity of the ligaments by fully trained surgeons (MND).

#### 2.5.2. Knee Muscle Strength

Two-minute rest interval was given between warm-up and testing. Before isokinetic measurement, all patients participated in a general body warm-up. The warm-up consisted of 5 minutes of submaximal exercise for lower extremity on a Biodex® System Pro3 (Biodex Corp. Shirley NY, USA) at 60°/sec. The same measurement protocol was reproduced for each of the testing sessions.

The isokinetic concentric peak torque of the knee muscles was determined bilaterally at an angular velocity 30°/s and 60°/s used for slow speeds, 180°/s for medium speed, and 330°/s used for a high speed by using a Biodex® System Pro3 isokinetic dynamometer. Tests were performed at 90° to 0° flexion for the knee. The order of the evaluation between the right and left sides of the knee was determined randomly. The monitor was placed in such a way to provide visual feedback to the patients. Three maximal repetitions at all velocity were performed with 5-minute rest between the tests. Standardized verbal instructions and encouragement were given.

The isokinetic test for the knee muscles was performed with the patients seated at 70° hip flexion (from the supine position) and the knee angle at 90° flexion [14]. The knee joint articulation axis was aligned to the dynamometer mechanic arm lever axis. The length of the dynamometer arm, which was fastened to the distal portion of the tibia by a Velcro strap, was altered according to the length of the leg of the patient.

#### 2.5.3. Knee Joint Position Sense

Proprioceptive status was assessed by joint position sense (JPS) using an active angle reproduction (AAR) technique with eyes closed [15], using the Biodex® System Pro 3 isokinetic dynamometer. During the AAR detection, patients were eyes closed and had their head turned away, looking at the opposite side of the dynamometer. The actual angle achieved by the patient and its difference from the target angle was recorded from the on-screen goniometer. When patients felt they had reached the target angle, they pressed the stop button and the angle was recorded; they were not permitted to correct the angle. This process was repeated six times for each target angle. The midrange angles were selected in an attempt to maximize sensory input from muscle proprioceptors. We decided that if a patient was 5° or more away from their target angle, the proprioceptive deficit was accepted [16].

The knee was moved from a 90° flexion starting position passively to each of the target angles of 75°, 45°, and 15° (with 15° close to full extension). The leg was held there for 10 s for the patient to memorize the position and then returned to 90° knee flexion. After a pause of 5 s, the patient moved the lower limb by active contraction at an angular velocity approximating 2°/s and stopped when the patient perceived that the target angle had been reached.

The mean of the six trials was taken for each patient at each angle and used to calculate the difference between the actual angles achieved and the target angles [17]. The absolute error of the total of six readings was taken. The absolute error is the difference between the actual angles relative to the target angle; this has no directional bias. The reliability for AAR testing was examined in our facility on 11 healthy controls having ICC^2,1^ of 0.716 (SEM 4.5) with eyes open and ICC^2,1^ 0.404 (SEM 3.87) with eyes closed.

#### 2.5.4. Functional Level

One-legged hop test was used to determine the functional level of patients. *The one-legged hop test* has good reliability and its intraclass correlation coefficients range from 0.97 to 0.99 [18]. Patients were asked to hop as far as possible from a predetermined line and to land on the same leg. The three trials were collected from both sides. The normal limb was tested first, followed by the reconstructed limb [19].

### 2.6. Statistical Analysis

The normality of the distribution of the data was investigated by Kolmogorov–Smirnov testing with alpha set at 0.05. This testing confirmed that the data were normally distributed and that further statistical analyses using the parametric testing would be appropriate. All data were analyzed with the Statistical Package for the Social Sciences (SPSS®) version 21.0 (SPSS Inc, Chicago, Illinois, USA). A comparison between the groups was performed using an independent *t*-test for age, body weight, and body height to determine whether the groups presented similar demographics. The paired samples *t*-test was used to compare operative and nonoperative sides of the patients. The independent samples *t*-test was used to compare for the data of muscle strength, joint position sense, and the single-legged hop test between the operative sides of the patients in groups. Statistical significance was set for all testing at *p* < 0.05.

## 3. Results

### 3.1. Recruitment and Sample Size

Thirty-two of the 57 patients completed the original study. The Consolidated Standards of Reporting Trials (CONSORT) table depicts patient flow throughout the study (see Table 2). The distributions of all numerical data were normal. There were no significantly differences in age (*F* = 0.220, *p*=0.64), height (*F* = 0.002, *p*=0.97), and body weight (*F* = 0.000, *p*=0.98) between groups.

The mean age was 29.35 ± 9.71 years (range of 16–53 years), the mean height was 174.47 ± 8.26 cm (range of 162–192 cm), and the mean weight was 75.29 ± 12.21 kg (range of 53–95 kg) of the patients in Group I. The mean age was 31.60 ± 8.45 years (range of 23–53 years), the mean height was 177.07 ± 7.24 cm (range of 168–188 cm), and the mean weight was 81.27 ± 12.31 kg (range of 63–105 kg) of the patients in Group II.

### 3.2. Outcomes of Subjective Parameters

The pivot shift, anterior drawer, and valgus stress tests of the patients were normal in both groups. Additionally, there was no lack of the knee flexion and extension range of motion of the patients.

### 3.3. Changes in Muscle Strength, Proprioception, and Hop Test Length

There were significant differences in muscle strength of the quadriceps and hamstring at 30°/s angular speed and in hop test between the operative and nonoperative sides in both groups (*p* < 0.05) (see Table 3). There were no differences in muscle strength of the quadriceps and hamstring at 60°/s, 180°/s, and 330°/s between the operative and nonoperative sides in Group I (*p* > 0.05). There were significant differences in muscle strength of the quadriceps (*p* < 0.001) and hamstring (*p*=0.01) at 60°/s between the operative and nonoperative sides in Group II (see Table 3). There was no difference in the knee joint position sense at 15°, 45°, and 75° between the operative and nonoperative sides in both groups (*p* > 0.05) (see Table 3). There was a difference in the hop test length between the operative and nonoperative sides in both groups (*p* < 0.05) (see Table 3).

There were significant differences in muscle strength of the quadriceps (*p*=0.01) and hamstring (*p*=0.01) at 330°/s and joint position sense at 75°, 45°, and 15° between the operative sides of the patients (*p* < 0.05) in Group I and Group II (see Table 4). There was no difference in the hop test length between the operative sides of the patients in both groups (*p* > 0.05) (see Table 4).

## 4. Discussion

The most important finding of the present study was that joint position sense at 15°, 45°, and 75° knee flexion of the patients in neuromuscular control exercises group was better than that in the standard rehabilitation group. Secondly, there was no difference in muscle strength between the groups at the lower and medium angular speeds (at 30°/s, 60°/s, and 180°/s). At the higher angular speed (at 330°/s), there was a difference between groups. Additionally, the pivot shift, anterior drawer, and valgus stress tests of the groups were similar. This is the first study to compare the neuromuscular control exercises program and standard rehabilitation program for patients with ACL-R using tibialis anterior tendon graft.

The autografts are commonly used for good functional outcomes [20] while donor muscle deficits of the hamstring and patellar tendon autografts are also well known [21]. Although allografts have potential advantages such as the absence of donor site morbidity, shorter operative times, improved cosmetics, and easier rehabilitation over autografts in ACL-R [22–24], functional scores of the ACL-R with a single loop freeze-dried irradiated tibialis anterior allograft were found similarly in four-strand hamstring autograft in nonathletic patients [25].

There is limited evidence on functional outcomes of the ACL-R with tibialis anterior allograft in the literature [22–25]. To our knowledge, limited number of studies evaluated only functional and subjective outcomes (IKDC and knee laxity) after the ACL-R using tibialis anterior allograft without the measurement of the muscle strength, hop distance, and proprioception. In the present study, clinical assessments, quadriceps and hamstring muscle strength, hop test, and proprioception of the knee joint were assessed. Additionally, outcomes of the neuromuscular control exercises and standard rehabilitation protocols were compared.

To the best of our literature research, we could not identify any previous study that evaluated the strength of the knee muscles, hop distance, and proprioception in patients with ACL-R using tibialis anterior allograft. Previous studies showed that intensive/accelerated rehabilitation program should improve quadriceps strength after ACL reconstruction using autografts, but it may not fully recover to preinjury level [6, 7, 26, 27]. Although muscle strength of the operative side of the patients was still lower than that of the nonoperative side in all groups, there was no considerable difference in the quadriceps and hamstring muscle strength between the groups at the lower and medium angular speeds. The standard program was found to be more effective in reducing the difference in endurance while the neuromuscular control program was found to be more effective in reducing the difference in strength between the operated and other knees. All patients included in the present study were sedentary. Positive effects on physical performance, muscle strength, functional level, and proprioception in sedentary patients who underwent neuromuscular control exercises program may be less than those in professional athletes.

It is emphasized that weight-bearing and postural/proprioceptive exercises would help to improve joint stability and proprioception following treatment in patients with ACL-R [27]. In the present study, decided that if a patient was 5° or more away from their target angle, the proprioceptive deficit was accepted. There were no proprioception deficits in patients. Additionally, joint position sense at 75°, 45°, and 15° of the operative side of the patients in Group I was found better than that of the operative side of the patients in Group II. Improving the proprioceptive sense is critical to prevent secondary injuries. Results of the proprioceptive sense could be explained by the specific exercises in the program with neuromuscular control exercises, which were designed to control lower extremity alignment and to help weight-bearing during the functional activities.

The primary limitation of the present study was that nonathletic patients who underwent ACL-R using tibialis anterior tendon allograft were included. The neuromuscular control exercises program should be performed in different grafts and professional athletes. Second limitation was that biomechanical and radiological evaluation was not performed. Biomechanical and radiological studies are required to determine the effects of the rehabilitation program on tunnel enlargement, gait and movement pattern, and graft healing. The third limitation of the present study was the 2-year follow-up. Long-term results of the program should be needed. Lastly, although the patients were frequently followed up, all patients were followed up with a home exercise program. Home exercises follow-up schedule was used while there is a need for studies where the exercises will be followed under the observation by physiotherapists.

Patient age and activity level, graft types, and rehabilitation protocols may contribute significantly to the outcome. The reason why these results are similar can be explained by the fact that the patients were sedentary and the exercises were followed by home exercises program.

Considering our results from muscle strength, proprioceptive sense, and functional test, the program with neuromuscular control exercises seems to be a reasonable and preferable option for nonathletic patients who undergo primary ACL-R with tibialis anterior allograft. Clinical relevance of the present study is that the program with neuromuscular control should be used to improve muscle strength and proprioceptive sense after ACL-R with tibialis anterior allograft.

## 5. Conclusion

The neuromuscular control exercises program was found to be more effective in reducing the difference in strength, while the standard program was found to be more effective in reducing the difference in endurance between the operated and other knees. This study revealed that neuromuscular control exercises should also be used to improve knee proprioception sense following ACL-R. Future studies might evaluate and compare the effects of the neuromuscular control exercises rehabilitation program for different grafts and professional athletes.

## Figures and Tables

**Table 1:** Neuromuscular control exercises program.

1^st^–3^rd^ days	*Mobilization*: tolerated weight-bearing mobilization with crutches
	*Exercises*:
	Quadriceps isometric setting *with towel under the heel*, straight leg raising (SLR) with full knee extension (with weights at ankle), raising the leg above the ground 40–50 cm in 10 seconds, holding for 10 seconds, and slowly lowering in 40 seconds
	*Restriction*: avoid active terminal knee extension (30° to full extension)

3^rd^ days-3^rd^ week	*Range of motion*: 0 to 90° flexion
	*Mobilization*: tolerated weight-bearing mobilization w/wo crutches
	*Exercises*:
	Quadriceps isometric setting with towel under the heel, SLR with full knee extension (with weights at ankle), raising the leg above the ground 40–50 cm in 10 seconds, holding for 10 seconds, and slowly lowering in 40 seconds, and heel slides (at 0–90° flexion)
	*Restriction*: avoid active terminal knee extension (30° to full extension)

3^rd^–6^th^ week	*Range of motion*: 0 to 120° flexion
	*Mobilization*: tolerated to full weight-bearing mobilization with knee brace
	*Exercises*:
	Single leg stance, balance reach leg and balance reach arm exercises, lunges (all directions), step-up (all directions) on other side of the patients, and bilateral squat
	*Restriction*: avoid active terminal knee extension (30° to full extension)

6^th^–12^th^ week	*Exercises:*
	Single leg stance, balance reach leg and balance reach arm exercises, lunges (all directions), step-up (all directions), step-down (all directions), one-legged squat, box heel touches (all directions), and single leg pelvic bridge on operated side of the patients, and ball exercises during athletic position

12^th^–24^th^ week	*Exercises*:
	Single leg straight leg dead lift, sumo squat, and weights added to all exercises
	Special stair exercises: (stair should be 18 cm in height and 30 cm in depth)
	*Explanation*:
	Stair exercise (1): stand behind a stair. While one foot on the ground, put the other foot on the stair and flex the knee about 45° flexion. Raise the body to full knee extension at one leg in 60 seconds and slowly lowering the body to 45° knee flexion in 60 seconds. During the exercises, the patient should control his/her lower leg to keep straight (keep away from varus/valgus)
	Stair exercise (2): stand on a stair. Lower the body at one leg in 60 seconds and slowly turn and raise the body in 60 seconds During the exercises, the patient should control his/her lower leg to keep straight (keep away from varus/valgus)

To 9th month	(3) running program (started at the 13^th^ week)
	(4) jumping (multidirectional) (started at the 18^th^ week)
	(5) plyometrics and agility exercises (started at the 20^th^ to 24^th^ week)
	*Restriction*: return to sports is not allowed before 9 months after surgery

**Table 2:** Consolidated Standards of Reporting Trials (CONSORT) table.

1. Assessed for eligibility	*n* = 57	
2. Enrolment	*n* = 57	
	^*∗*^Exclusions: *n* = 17	
	Meniscal repair/meniscectomy/chondral repair: *n* = 13	
	Female patients: *n* = 4	
	Group I	Group II
3. Randomized	*n* = 20	*n* = 20
4. Lost to follow-up	*n* = 3	*n* = 5
5. 6 months after surgery	*n* = 17	*n* = 15
6. 24-month follow-up	*n* = 17	*n* = 15

Group I: neuromuscular control exercises were performed; Group II: standard rehabilitation program was performed; *n*: number of patients.

**Table 3:** Descriptive and compared values of muscle strength, joint position sense, and hop tests obtained for the operated side and the other side of patients.

		Group I (*n* = 17)			Group II (*n* = 15)		
		Operated side (mean ± SD)	Other side (mean ± SD)	*p*	Operated side (mean ± SD)	Other side (mean ± SD)	*p*
Quadriceps muscle strength (Nm)	30°/s	138.71 ± 35.7	162.24 ± 49.12	**0.01** ^*∗*^	138.67 ± 34.05	160.27 ± 39.05	**0.001** ^*∗*^
	60°/s	128.06 ± 33.47	140.94 ± 30.53	0.19	120.13 ± 36.86	147.00 ± 37.16	**0.001** ^*∗*^
	180°/s	88.82 ± 24.09	93.53 ± 24.77	0.39	94.93 ± 40.88	98.73 ± 26.61	0.60
	330°/s	57.00 ± 9.50	58.82 ± 18.47	0.64	62.80 ± 25.36	68.20 ± 26.15	0.28

Hamstring muscle strength (Nm)	30°/s	98.88 ± 25.66	112.94 ± 28.60	**0.01** ^*∗*^	96.73 ± 23.64	114.20 ± 29.88	**0.02** ^*∗*^
	60°/s	99.00 ± 20.46	101.18 ± 20.93	0.62	92.13 ± 25.00	109.20 ± 26.07	**0.01** ^*∗*^
	180°/s	79.41 ± 16.62	82.00 ± 18.37	0.62	79.07 ± 27.59	85.20 ± 23.12	0.45
	330°/s	73.24 ± 11.24	74.41 ± 11.78	0.77	76.13 ± 20.49	77.67 ± 24.73	0.74

JPS (°)	75°	74.55 ± 1.54	74.61 ± 2.98	0.93	75.85 ± 3.77	76.92 ± 3.42	0.22
	45°	45.63 ± 2.94	45.19 ± 4.11	0.64	47.32 ± 6.05	47.18 ± 5.84	0.92
	15°	15.44 ± 1.74	15.78 ± 2.40	0.64	16.95 ± 3.13	17.07 ± 2.49	0.93

Hop test (cm)	141.37 ± 34.35	156.12 ± 24.04	**0.01** ^*∗*^	146.53 ± 19.56	157.31 ± 19.53	**<0.001** ^*∗*^	

^*∗*^Paired sample *t*-test. JPS: joint position sense; Group I: neuromuscular control exercises were performed; Group II: standard rehabilitation program was performed; SD: standard deviation; *n*: number of patients.

**Table 4:** Descriptive and compared values of muscle strength, joint position sense, and hop tests obtained for the operated side of the patients in Group I and the operated side of patients in Group II.

		Group I (*n* = 17)		Group II (*n* = 15)		*F*	*p*
		Operated side (mean ± SD)	Other side (mean ± SD)	Operated side (mean ± SD)	Other side (mean ± SD)		
Quadriceps muscle strength (Nm)	30°/s	138.71 ± 35.7	162.24 ± 49.12	138.67 ± 34.05	160.27 ± 39.05	0.362	0.55
	60°/s	128.06 ± 33.47	140.94 ± 30.53	120.13 ± 36.86	147.00 ± 37.16	0.037	0.85
	180°/s	88.82 ± 24.09	93.53 ± 24.77	94.93 ± 40.88	98.73 ± 26.61	2.389	0.13
	330°/s	57.00 ± 9.50	58.82 ± 18.47	62.80 ± 25.36	68.20 ± 26.15	10.138	**0.01** ^*∗*^

Hamstring muscle strength (Nm)	30°/s	98.88 ± 25.66	112.94 ± 28.60	96.73 ± 23.64	114.20 ± 29.88	0.371	0.55
	60°/s	99.00 ± 20.46	101.18 ± 20.93	92.13 ± 25.00	109.20 ± 26.07	0.371	0.55
	180°/s	79.41 ± 16.62	82.00 ± 18.37	79.07 ± 27.59	85.20 ± 23.12	1.565	0.22
	330°/s	73.24 ± 11.24	74.41 ± 11.78	76.13 ± 20.49	77.67 ± 24.73	7.102	**0.01** ^*∗*^

JPS (°)	75°	74.55 ± 1.54	74.61 ± 2.98	75.85 ± 3.77	76.92 ± 3.42	28.990	**0.001** ^*∗*^
	45°	45.63 ± 2.94	45.19 ± 4.11	47.32 ± 6.05	47.18 ± 5.84	7.899	**0.01** ^*∗*^
	15°	15.44 ± 1.74	15.78 ± 2.40	16.95 ± 3.13	17.07 ± 2.49	9.014	**0.01** ^*∗*^

Hop test (cm)	141.37 ± 34.35	156.12 ± 24.04	146.53 ± 19.56	157.31 ± 19.53	2.323	0.14	

^*∗*^Independent sample *t*-test. JPS: joint position sense; Group I: neuromuscular control exercises were performed; Group II: standard rehabilitation program was performed; SD: standard deviation; *n*: number of patients.

## Data Availability

The data used to support the findings of this study are available from the corresponding author upon request.
